# Molecular Aspects of COVID-19 Differential Pathogenesis

**DOI:** 10.3390/pathogens9070538

**Published:** 2020-07-06

**Authors:** Hussin A. Rothan, Arpan Acharya, St Patrick Reid, Mukesh Kumar, Siddappa N. Byrareddy

**Affiliations:** 1 Department of Biology, College of Arts and Sciences, Georgia State University, Atlanta, GA 30303, USA; 2 Department of Pharmacology and Experimental Neuroscience, University of Nebraska Medical Centre, Omaha, NE 68198, USA; arpan.acharya@unmc.edu; 3 Department of Pathology and Microbiology, University of Nebraska Medical Center, Omaha, NE 68198, USA; patrick.reid@unmc.edu; 4 Department of Genetics, Cell Biology, and Anatomy, University of Nebraska Medical Centre, Omaha, NE 68198, USA; 5 Department of Biochemistry and Molecular Biology, University of Nebraska Medical Centre, Omaha, NE 68198, USA

**Keywords:** SARS-COV-2, COVID-19, differential pathogenesis, comorbidity, cardio-metabolic disease

## Abstract

In the absence of therapeutic interventions, and a possible vaccine candidate, the spread of COVID-19 disease and associated fatalities are on the rise. The high mutation frequency in the genomic material of these viruses supports their ability to adapt to new environments, resulting in an efficient alteration in tissue tropism and host range. Therefore, the coronavirus’ health threats could be relevant for the long-term. The epidemiological data indicate that age, sex, and cardio-metabolic disease have a significant impact on the spread and severity of COVID-19. In this review, we highlight recent updates on the pathogenesis of SARS-CoV-2 among men and women, including children. We also discuss the role of the cellular receptors and coreceptors used by the virus to enter host cells on differential infection among men, women, and cardio-metabolic patients.

## 1. Introduction to SARS-COV-2 Infection

Infection with severe acute respiratory syndrome coronavirus-2 (SARS-COV-2) that appeared in December 2019 (COVID-19) in China has become a global public health threat [1]. There are no effective vaccines or specific antiviral drugs available to manage COVID-19 disease. Generally, coronaviruses pose severe threats to human health and economic stability worldwide. The high mutation frequency in the genomic material of these viruses supports their ability to adapt to new environments, resulting in an efficient alteration in tissue tropism and host range [2,3,4]. Recent findings support that the binding affinity of SARS-COV-2 envelop spikes to the cellular ACE2 receptor is 10–20 times higher than SARS-COV-1, facilitating the high transmission and infectivity of SARS-COV-2 in humans [5].

Based on the genetic sequence identity and the phylogenetic reports, SARS-COV-2 is significantly different from SARS-COV-1, and it can thus be considered a novel beta coronavirus that infects humans. SARS-COV-2 most likely developed from the bat origin coronaviruses and was transmitted to humans by yet unknown methods [1]. Coronaviruses (CoVs) are enveloped positive-sense and single-stranded RNA viruses that belong to the Coronaviridae family and Nidovirales order [6]. The CoVs are classified into four genera: alpha-CoVs (α-CoVs), beta-CoVs (β-CoVs), gamma-CoVs (γ-CoVs), and delta-CoVs (δ-CoVs) [7]. CoVs are spherical in the structure, having a diameter that ranges from 80 to 220 nm [8]. The name coronavirus (CoVs) originates from solar corona since spike glycoproteins of CoVs project from their envelop as a solar corona-like appearance [9]. The genomic RNA scale varies from 26 to 32 kb long, which is the largest among the currently known RNA viruses [10,11]. The RNA genome consists of a 5′ cap and 3′ poly-A tail. About 2/3 (20 kb) of the genome encompasses open reading frames (ORFs) 1a and 1b, which code for nonstructural proteins, whereas the remaining 1/3 (10 kb) codes for structural proteins including spike (S), envelop (E), membrane (M), and nucleocapsid (N) proteins [8].

There are about a hundred species of CoVs circulating in numerous animals, including bats, camels, pigs, horses, dogs, cats, birds, rodents, rabbits, ferrets, minks, snakes, and other wildlife animals [12]. Bats are the foremost common natural reservoirs of CoVs, as is the case for many other exotic zoonotic viruses such as Ebola, rabies, Hendra, and Nipah [13]. The homologs recombination between the structural protein of coronaviruses originating from diverse hosts is responsible for their cross-species transmission, which frequently spills over to humans [2]. Human coronaviruses (HCoVs) like HCoV-229E, HCoV-OC43, HCoV-NL63, HCoV-HKU1, severe acute respiratory syndrome (SARS)-CoV, and middle east respiratory syndrome (MERS)-CoV generally cause respiratory and enteric diseases [14]. The disease manifestation of HCoVs varies widely from the common cold to pneumonia-like diseases that may trigger acute respiratory distress syndromes (ARDS). In late 2002, SARS-CoV emerged in south China after a cross-species transmission from its natural host bats, spread across the globe, infected 8098 people, of which 774 succumbed to the disease [15]. SARS-CoV enters human cells using ACE2 receptors and causes upper respiratory tract infection, which eventually spreads to the lower respiratory tract, causing severe pulmonary injury and dysfunction immune response.

On the other hand, the MERS-CoV outbreak, first reported in June 2012 from the Kingdom of Saudi Arabia, stemmed from a cross-species transmission from camels to humans. Up to 2019, 2040 people were infected across 27 countries with MERS-CoV, of which 712 died [16]. MRRS-CoV uses dipeptidyl peptidase 4 (DPP4) as a receptor to enter the host cell and causes lower respiratory tract infection and damage to the innate and adaptive immune system, resulting in a cytokine storm. Other organs like the kidneys, intestine, and liver are also susceptible to MERS-CoV infection.

As per the Johns Hopkins University and Medicine coronavirus resource center, as of 6 June 2020, 6.98 million people have tested positive globally for SARS-CoV-2, of which 401,933 patients have died (https://coronavirus.jhu.edu/map.html). SARS-CoV-2 belongs to β-CoVs [17], which has around 96% sequence similarity to a CoVs isolated from bats in Hubei province named RaTG13, and it is speculated that bats maybe its natural reservoir [18]. Another study reports a 91.02% sequence similarity between SARS-CoV-2 and a virus found in dead Malayan pangolins, indicating pangolins may be an intermediate reservoir [19]. The symptoms of COVID-19 include sore throat, dry cough, fever, shortness of breath, fatigue, muscle aches, runny nose, and diarrhea [20]. SARS-CoV-2 infection also causes several neurological manifestations that include, but are not limited to, headache, nausea, vomiting, loss of taste and smell, acute cerebrovascular diseases, Guillain-Barré syndrome, and impaired consciousness [21].

SARS-COV-2 targets the lower respiratory airway, causing severe acute syndrome with increasing death cases. Susceptibility to infection with SARS-COV-2 and disease severity varies amongst individuals, factoring age, sex, and health conditions. Current clinical data suggest that geriatric people and people with comorbidities such as cardiovascular disease, diabetes, chronic lung disease and hypertension appear to develop acute severe respiratory distress syndrome (ARDS) compared with others [22,23]. Recent data from the SARS-CoV2 outbreak indicate that some of the critically ill COVID-19 patients develop “cytokine release syndrome,” resulting in a dysregulated hyperinflammatory response in the lungs and subsequently in the heart, kidneys, CNS, and other organs, eventually leading to multi-organ failure [24,25]. Recent evidence suggests that respiratory failure and stroke may be driven by arterial and venous thromboembolism and endothelial dysfunction [26,27,28,29]. Here, we discuss the epidemiology of the COVID-19 illness based on the patient groups and the severity of the infection. We also discuss the role of the cellular receptors and coreceptors in virus infectivity, comorbidity with cardio-metabolic syndromes, and the overlap between cardio-metabolic treatments and SARS-CoV-2 infection.

## 2. COVID-19 Illness in Children and Women

The epidemiological reports have shown that the death cases of COVID-19 illness are higher in healthy or older adults than children. A study showed that among 44,672 COVID-19 confirmed cases, only 549 (1.2%) were between 10–19 years old, and 416 (0.9%) were between 0–10 years [30]. About 4% of children were asymptomatic, 51% had a mild illness, and 39% had a moderate illness. Another study showed that the percentage of severe SARS-CoV-2 infection among children was 6% compared to 18.5% in adults [31]. Generally, infected children show milder to asymptomatic COVID-19 disease. Adults with severe COVID-19 disease suffer from deadly pneumonia and insufficient supply of oxygen throughout the body, as reported by several frontline clinicians.

On the other hand, clinical reports showed that the children are not susceptible to pneumonia caused by SARS-CoV-2 infection [23,32,33,34,35]. The considerable variation in SARS-CoV-2 infection between children and adults raises a question that could help understand the mechanism of COVID-19 disease pathogenesis. Although initial reports indicate that SARS-CoV-2 positive children only develop a mild disease, new updates from clinics report cases of “severe shock syndrome with hyper inflammation”, and “Kawasaki-like-disease” [36,37,38]. In late April 2020, The Royal College of Pediatrics from the UK reported systemic inflammatory response in a fraction of SARS-CoV-2 positive children with cardiac injury that have overlapping features with Kawasaki disease. Most of the children had a higher level of C-reactive proteins, D-dimers, troponin, and significant decreases in lymphocyte count [39]. In early May 2020, clinicians from New York City reported similar findings among a fraction of SARS-CoV-2 positive children [40]. Similarly, Paris reports indicate an increase in incidences of “Kawasaki-like multisystem inflammatory syndrome” among children, which may be related to SARS-CoV-2 [41]. All these findings indicate that all SARS-CoV-2 positive children should be carefully monitored for the development of systemic inflammatory responses and associated cardiac injury for better management of the disease.

Interestingly, the reports showed that the rate of SARS-CoV-2 infection is higher in men than women, and the severity of the illness is much higher in men. The percent of disease fatality in men is 2.8%, while in women, it is 1.7% [42,43]. An early study from China showed that of the 99 patients with COVID-19 pneumonia, the combined average age of the patients was 55·5 years, while the average age for men was 67 years, and for women, it was only 32 years [44]. Another report showed that of 55,924 COVID-19 confirmed cases reported in February 2020, the men comprised 51.1%, women comprised 21.6%, and the median age was 51 years [43]. The differential pathogenesis of SARS-COV-2 among different groups requires further investigations that focus on the disease’s molecular mechanism. Understanding the mechanism of COVID- 19 pathogenesis would help in the development of an efficient cure.

## 3. The Potential Role of Viral Cellular Receptors in the Severity of COVID-19 Illness

### 3.1. The Role of Angiotensin-Converting Enzyme-2 (ACE2)

Angiotensin-converting enzyme-2 (ACE2) represents the primary SARS-CoV-2 entry receptor, and its physiological role is crucial in the progress of COVID-19 illness. Lung epithelial cells that express the ACE2 receptor on the cell membrane are the primary target of coronaviruses. Binding of the virus spikes to the receptor-binding domain of the ACE2 initiates SARS-CoV entry into target cells [45,46]. Amino acid sequence data shows similarities between SARS-CoV-2 and SARS-CoV-1, and the recent reports strongly suggest that the entry of SARS-CoV-2 to the host cells is via the ACE2 receptor [45,47]. It is important to note that the ACE2 gene is mapped on the human X chromosome (Xp22) [48,49], and the female hormone 17β-estradiol increases the expression levels of ACE2 protein, as investigated in ovariectomized female rats [50]. Furthermore, a significantly higher ACE2 expression was detected in older females than male rats [51], and such high expression levels of ACE2 could be crucial in preventing kidney injury in the experimental hypertensive model [50].

Previous studies on SARS-CoV-1 reported that the binding of viral spike (S) protein to ACE2 downregulates the expression of ACE2, resulting in a diminished protective role of ACE2 and, subsequently, acute respiratory failure [52]. Furthermore, ACE2 expression is dramatically reduced with aging in both genders [51]. The levels of ACE2 expression, which could be sex- and age-dependent, have a protective role against lung and kidney injuries that could impact the severity of COVID-19 illness in male vs. females and old vs. young individuals. The initiation of SARS virus infection is ACE2-dependent, and after that, there are ACE2-independent pathways for cell-to-cell virus transmission [53]. Cell-to-cell spreading has a significant impact on virus infection and pathogenesis. The ACE2 downregulation due to virus infection, as mentioned above, contributes to lung and kidney injuries. We hypothesize that the reduction in ACE2 levels in men is higher than females, which would explain the observed increases in disease severity.

### 3.2. The Role of the Coreceptor TMPRSS2

**Figure 1:**
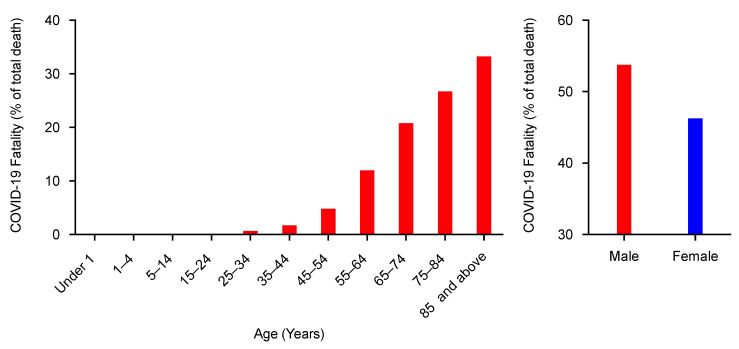
Age and gender distribution of deaths due to coronavirus disease 2019 (COVID-19) in the United States as of 10 June 2020. The data are calculated based on the total number of deaths (95,608) as of 10 June 2020, which was received and coded by the National Center for Health Statistics. Data source—Centers for Disease Control and Prevention: National Center for Health Statistics. https://www.cdc.gov/nchs/nvss/vsrr/COVID19/.

ACE2 receptor is coexpressed with TMPRSS2, a cellular transmembrane protease that cleaves the S protein of SARS-CoV-1 and SARS-CoV-2 into two fragments: S1, which is essential for virus attachment, and S2, for virus fusion into the target cells [47,54,55,56]. TMPRSS2 protein is expressed in many tissues, including the lungs, primarily in the epithelial cells [57,58]. The expression levels of TMPRSS2 protein are regulated by levels of androgen and androgen receptors [58,59,60,61], suggesting sex-related expression levels of TMPRSS2 protein. Both women and children have a lower level of androgen and androgen receptors than men, and therefore, TMPRSS2 could play a potential role in the severity of COVID-19 pathogenesis in men. This pattern is supported when we take the total deaths in the United States due to COVID-19 and consider the breakdown of age percentages and sex distribution (Figure 1) [62]. The percentage of deaths in male patients is higher compared to female COVID-19 patients. Thus, it could be possible that the expression levels of ACE2 and TMPRSS2 impact virus infectivity and pathogenesis among different groups of individuals, considering the variation in the expression levels in older men compared to the women and children. 

## 4. Comorbidity of COVID-19 Illness and Cardio-Metabolic Syndromes

The severity and mortality of SARS-CoV-2 infection are positively correlated with the comorbidity of lung disease, diabetes, cardiovascular diseases and cerebrovascular diseases. A study reported that 173 patients from 1099 confirmed cases of COVID-19 had comorbidities of hypertension (23.7%), diabetes mellitus (16.2%), heart diseases (5.8%), and cerebrovascular disease (2.3%), and all of the 173 patients showed severe COVID-19 illness [63]. Another report showed that of a group of 52 SARS-CoV-2-infected patients, 32 patients died with comorbidities of diabetes and cerebrovascular diseases (22%) [64]. Another study reported that of 140 severe cases of COVID-19, about 30% of the patients experienced chronic hypertension, and 12% had diabetes [65]. The susceptibility of cardio-metabolic patients to develop severe COVID-19 illness and the high mortality rate could be linked to the ACE2 function during SARS-CoV-2 infection and the cardio-metabolic treatments that may interfere with ACE2–virus interaction.

## 5. SARS-CoV-2 Infection and ACE2 Expression in Cardio-Metabolic Patients

ACE cleaves angiotensin (Ang) I to form Ang II within the renin-angiotensin system (RAS). Ang II has been recognized as the primary active peptide cleaved by ACE2, a homolog of ACE, to form Ang (1–7). ACE2 has high catalytic efficiency, suggesting an essential role in preventing Ang II accumulation while enhancing Ang-(1–7) formation [66,67]. ACE2 alterations have been described in experimental models of hypertension and diabetic kidney disease, and ACE2 levels were found to be decreased in the setting of hypertension [68,69,70,71]. ACE2 expression is dramatically reduced with aging in both genders and young adult vs. old [51]. Thus, ACE2 overexpression improves pancreatic islet-cell function, cardiovascular health, blood pressure, and the renal protective arm of the RAS [72]. ACE2 has a therapeutic effect on diabetes, cardiovascular conditions, kidney disease, and several other conditions in which the overactivity of Ang II is undesirable. Previous studies on SARS-COV-1 reported that the binding of viral S protein to ACE2 downregulates the expression of ACE2, resulting in a diminished protective role of ACE2 and, subsequently, acute respiratory failure [52]. Downregulation or malfunction of ACE2 leads to the accumulation of Ang II, resulting in a significant reduction in insulin secretion from the pancreas and the glomerular filtration rate in the kidney [73]. A previous study by Wysocki et al. tested whether a soluble human recombinant ACE2 (rACE2) may be used to decrease ANG II and increase ANG (1–7) levels in plasma and tissues and whether rACE2 may be used to prevent ANG II-induced hypertension in mice. Interestingly, this study found that rACE2 infusion induced a dose-dependent increase in serum ACE2 activity but had no effect on kidney or cardiac ACE2 activity [67]. However, the role of indigenous or exogenous circulating soluble ACE2 on the progression of COVID-19 still needs further investigation.

Aging decreased expression of the ACE2, which also leads to the accumulation of Ang II levels, affecting other body organs like the heart, pancreas, and kidneys. These factors suggest that treatment with ACE2-activating compounds could enhance hypertension and diabetic kidney disease during infection. The impact of ACE2 activators/inhibitors on the COVID-19 illness requires urgent investigation. The American Heart Association, the Heart Failure Society of America, the American College of Cardiology, and the Council on Hypertension of the European Society of Cardiology have urged that the current therapies should be continued at this point of COVID-19 illness as the withdrawal of the ARB therapies may be unwise since there is no clinical evidence for the interaction of ARB therapies and COVID-19 illness [74,75]. Recent reports have supported these recommendations, indicating that the results of clinical studies showed no evidence that ACE inhibitors or ARBs affected the risk of COVID-19, and there is no potentially harmful association of ACE inhibitors or ARBs with in-hospital deaths caused by COVID-19 illness [76,77,78].

We conclude that the variations in the expression levels of SARS-CoV-2 receptors and co-receptors, due to physiological and co-morbidity conditions, could impact the differential pathogenesis of COVID-19. Further experimental studies are required to investigate the impact of genetic variations in ACE2 receptors and circulating soluble ACE2 on the severity of COVID-19 illness.
